# Modifiable Risk Factors for Developing Diabetes Among Women With Previous Gestational Diabetes

**Published:** 2006-12-15

**Authors:** Shumei Yun, Nisreen H Kabeer, Bao-Ping Zhu, Ross C Brownson

**Affiliations:** Missouri Department of Health and Senior Services, Division of Community and Public Health; Division of Community and Public Health, Missouri Department of Health and Senior Services, Jefferson City, Mo; Division of Community and Public Health, Missouri Department of Health and Senior Services, Jefferson City, Mo; Department of Community Health and Prevention Research Center, School of Public Health, Saint Louis University, St Louis, Mo

## Abstract

**Introduction:**

Gestational diabetes mellitus (GDM) affects approximately 2% to 4% of all pregnant women in the United States each year. Women who have had GDM are at high risk for developing nongestational diabetes. The objective of this study was to assess the prevalence of modifiable risk factors for developing diabetes among women with previous GDM only.

**Methods:**

Cross-sectional data for nonpregnant women from the 2003 Behavioral Risk Factor Surveillance System were used to estimate and compare the prevalence of modifiable risk factors among three groups: nonpregnant women with previous GDM only, nonpregnant women with current diabetes, and nonpregnant women without diabetes.

**Results:**

In 2003, 7.6% of nonpregnant women aged 18 years and older in the United States had current self-reported physician-diagnosed diabetes, and 1.5% had previous GDM only. Compared with women without diabetes, women with previous GDM only had higher prevalence of no leisure-time physical activity (32.0% vs 25.7%), overweight (62.2% vs 49.0%), and obesity (29.4% vs 20.0%). After adjusting for sociodemographic variables, women with previous GDM only were more likely to have no leisure-time physical activity (prevalence odds ratio [POR], 1.4; 95% confidence interval [CI], 1.2–1.7) and more likely to be overweight (POR, 1.8; 95% CI, 1.6–2.2) or obese (POR, 1.7; 95% CI, 1.4–2.1), compared with women with no diabetes.

**Conclusion:**

Women with previous GDM are more likely to have modifiable risk factors for developing diabetes than women without diabetes. More attention to this issue is needed from health care providers and public health officials to encourage the promotion of healthy lifestyles during and after pregnancy.

## Introduction

Gestational diabetes mellitus (GDM) is defined as glucose intolerance that is first detected during pregnancy. In the United States, GDM affects approximately 2% to 4% of all pregnant women, or approximately 135,000 women each year (1). An increase in the prevalence of GDM has been reported among pregnant women enrolled in Kaiser Permanente, Colorado (KPCO). It was estimated that the prevalence of GDM among KPCO members doubled from 2.1% in 1994 to 4.1% in 2002 (2). Women who have had GDM are at high risk for developing nongestational diabetes. Research has shown that women with gestational diabetes have a 17% to 63% risk of developing nongestational diabetes within 5 to 16 years after index pregnancy (2-6). The National Institutes of Health's Diabetes Prevention Program (DPP) and the Finnish Diabetes Prevention Study showed that progression to type 2 diabetes among subjects with prediabetes is preventable through lifestyle interventions (7,8). Fifteen percent of the subjects in the two studies had a history of GDM, suggesting that prevention through lifestyle interventions might be similarly achievable in the GDM population.

Because of the significant health risks associated with type 2 diabetes, women with GDM should be informed by health care professionals about their risk for developing diabetes and about the potential of lifestyle modification in preventing diabetes. If women with previous GDM were well-informed of their risk and motivated to change their behaviors, a lower prevalence of behavioral risk factors would be expected among these women. However, the prevalence of behavioral and other modifiable risk factors for developing diabetes among women with previous GDM is not well documented.

The objectives of this study are to assess the prevalence of modifiable risk factors for developing diabetes among women in the United States with previous gestational diabetes and to compare this prevalence with the prevalence among women with and without diabetes. The study will help diabetes prevention and control programs to evaluate the burden of modifiable risk factors among women with previous GDM and the potential for prevention of diabetes among these women.

## Methods

### Data source

We used data from the 2003 Behavioral Risk Factor Surveillance (BRFSS) in this analysis. The BRFSS is a survey of health risk factors and is sponsored by the Centers for Disease Control and Prevention (CDC) (9). It is a standardized, random-digit–dialed telephone survey among the noninstitutionalized adult U.S. population carried out by health agencies in all 50 states and the District of Columbia. The primary purpose of the BRFSS is to provide state-specific estimates of the prevalence of self-reported behavioral risk factors that are associated with the leading causes of death and disability in the United States. Each participating state independently selects for interview a probability sample from adult residents aged 18 years and older in households with telephones. All states in a given year use an identical core questionnaire administered over the telephone by trained interviewers. We included data for nonpregnant women aged 18 years and older in the analysis. Pregnant women (n = 2835) were excluded from the analysis.

### Variable definitions

During the telephone interview, respondents were asked, "Have you ever been told by a doctor that you have diabetes?" Female respondents who answered yes were asked, "Was this only when you were pregnant?" Women who responded yes to the first question and no to the second question were classified as having current physician-diagnosed diabetes (*current diabetes*); women who answered yes to both questions were classified as having *previous GDM only*; and women who answered no to the first question were classified as not having physician-diagnosed diabetes (*no diabetes*). Women who have current diabetes may or may not have had previous GDM; however, the information was not collected in this survey.

The age groups were categorized into 18 to 44 years, 45 to 64 years, and 65 years and older. Race and ethnicity were combined into eight categories. Education level was defined as having less than a high school education, a high school or general equivalency diploma (GED), some college or technical school education, or a college or technical school degree. Annual household income was categorized into five levels: less than $15,000; $15,000 to $24,999; $25,000 to $34,999; $35,000 to $49,999; and $50,000 or more.

Smoking status was determined by the following two questions: "Have you smoked at least 100 cigarettes in your entire life?" and "Do you now smoke cigarettes every day, some days, or not at all?" A current smoker was defined as an individual who responded yes to the first question and "every day" or "some days" to the second question.

Body mass index (BMI) (weight [kg]/height [m^2^]) was calculated from self-reported weight and height. Individuals with a BMI of 25.0 kg/m^2^ or greater were considered overweight, and those with a BMI of 30.0 kg/m^2^ or greater were considered obese. Leisure-time physical activity was defined as participation in any physical activity or exercise such as running, calisthenics, golf, gardening, or walking for exercise during the past month, not including activity during a regular job. Meeting CDC's recommended levels of physical activity was determined by whether the respondent reported achieving sufficient moderate-intensity physical activity (30 minutes per day on 5 or more days per week) or vigorous physical activity (20 minutes or more per day on 3 or more days per week), or both. Fruit and vegetable consumption was determined by a series of six questions on how often the following were consumed: fruit juices such as orange, grapefruit, or tomato; fruit other than juices; green salad; potatoes (not including French fries, fried potatoes, or potato chips); carrots; and other vegetables. The CDC recommendation for fruit and vegetable consumption is five or more servings each day.

### Data analysis

We used STATA (StataCorp LP, College Station, Tex) for data analysis. To account for design effects, data were weighted to adjust for the unequal probability of selection, differential nonresponse, and possible deficiencies in the sampling frame. We used a design-based F test to compare the demographic distributions of the study population among diabetes groups (10). We generated prevalence estimates of modifiable risk factors by diabetes status. We used logistic regression to adjust for age, race and ethnicity, and education when we compared the prevalence of modifiable risk factors by diabetes status. We presented crude and adjusted prevalence odds ratios (PORs) and 95% confidence intervals (CIs).

The Institutional Review Board of the Missouri Department of Health and Senior Services reviewed the study protocol and determined it to be exempt.

## Results

Of the 158,746 women who participated in the 2003 BRFSS, we excluded 2835 pregnant women from this analysis. Of these pregnant women, 1.2% reportedly had diabetes at the time of the survey; an additional 3.4% had been diagnosed as having GDM in either the current pregnancy or a previous pregnancy. Of the 155,911 nonpregnant women aged 18 years and older included in this study, 12,677 (8.1%) reported current diabetes, and 2123 (1.4%) reported previous GDM only. We estimated that in 2003, 7.6% of nonpregnant women aged 18 years and older had self-reported diabetes in the United States; an estimated 1.5%, or 1.7 million, had previous GDM, but had not yet developed diabetes.

The compositions of age, race and ethnicity, education, and household income varied among the three diabetes groups. Compared with women without diabetes, women with previous GDM only were more likely to be younger, more likely to be Hispanic or non-Hispanic Asian, less likely to be non-Hispanic white, and were similar in levels of education and household income. Conversely, compared with women with no diabetes, women with current diabetes were more likely to be older, more likely to be non-Hispanic black, less likely to be non-Hispanic white, more likely to have a high school education or less, and more likely to have a household income of less than $25,000 (Table 1).

Table 2 shows that compared with women without diabetes, women with previous GDM only had significantly higher prevalence of no leisure-time physical activity (32.0% vs 25.7%), overweight (62.2% vs 49.0%), and obesity (29.4% vs 20.0%). Women with current diabetes had significantly higher prevalence of no leisure-time physical activity (41.6%), not meeting CDC physical activity recommendations (70.9%), overweight (80.5%), obesity (51.2%), and not consuming adequate fruit and vegetables (31.3%) compared with women with no diabetes or with previous GDM only.

After adjusting for sociodemographic variables, women with previous GDM only were more likely to have no leisure-time physical activity (POR, 1.4; 95% CI, 1.2–1.7), more likely to be overweight (POR, 1.8; 95% CI, 1.6–2.2), and more likely to be obese (POR, 1.7; 95% CI, 1.4–2.1) compared with women with no diabetes. Likewise, women with current diabetes were more likely to have no leisure-time physical activity (POR, 1.4; 95% CI, 1.3–1.5), more likely to not meet CDC physical activity recommendations (POR, 1.4; 95% CI, 1.3–1.6), and more likely to be overweight (POR, 3.4; 95% CI, 3.1–3.7) and obese (POR, 3.6; 95% CI, 3.4–3.9) compared with women with no diabetes. The PORs for not meeting CDC physical activity recommendations, current smoking status, overweight and obesity, and not consuming adequate fruits and vegetables were all higher for women with current diabetes than for women with previous GDM only.

## Discussion

Diet and exercise are regarded as important components in the prevention and self-management of diabetes (11). Appropriate diet and exercise can be effective in improving insulin sensitivity and glycemic control (10). Participants in the lifestyle intervention group of the DPP received counseling on diet, exercise, and behavior modification during the 3-year study, reducing their risk of developing diabetes by 58%. The study was conducted among individuals with impaired glucose tolerance; approximately 15% of DPP's study population was composed of women with previous GDM (7).

In this study, we found that women with previous GDM only were less active and more likely to be overweight or obese compared with women with no diabetes. Physical inactivity, overweight, and obesity are major modifiable risk factors for type 2 diabetes (12). Lifestyle modification — 150 minutes of moderate-intensity physical activity per week, a healthy diet, and moderate (5%–10%) weight loss — can prevent or postpone type 2 diabetes among people at high risk (7). Therefore, there is a large potential for preventing diabetes among women with GDM. To prevent diabetes, women with GDM should be informed about their risk of developing diabetes and be educated and motivated to change their behaviors.

Compared with women with no diabetes, women with current diabetes were less active, more likely to be overweight or obese, and less likely to consume adequate amounts of fruits and vegetables. In some categories, prevalence rates were striking. For example, we found that 70.9% of women with current diabetes were not meeting physical activity recommendations, and 80.5% of women with current diabetes were overweight. Additionally, PORs for physical activity, smoking status, overweight and obesity, and fruit and vegetable intake for women with current diabetes were higher than that for women with previous GDM only. Therefore, women with diabetes are more likely to have these modifiable risk factors than women with previous GDM only. It was estimated that about 10% to 31% of parous women with current diabetes had previous GDM (13). Therefore, we could speculate that women with current diabetes who have had previous GDM may be more likely to have one or more of the modifiable risk factors than women with similar sociodemographic characteristics and previous GDM only.

Smoking may be an independent risk factor for diabetes (14,15) and also increases the risk of cardiovascular disease among people with diabetes (16). This study found that women with diabetes or previous GDM are equally likely to be current smokers compared with those with no diabetes. Therefore, diabetes education should include smoking cessation components.

Based on the findings in this study, we recommend that women with previous GDM should be educated and motivated to change their lifestyle to prevent diabetes; women with diabetes should also be educated about the importance of a healthy lifestyle in preventing diabetes-related complications. Diabetes self-management courses with appropriate information on lifestyle modification should be offered to all people with diabetes.

We estimated that approximately 1.5%, or 1.7 million, nonpregnant women aged 18 and older had previous GDM in the United States in 2003, not including those who had previous GDM and had progressed to diabetes. Among 2835 surveyed pregnant women, 3.4% had been diagnosed as having GDM in either a current pregnancy or a previous pregnancy. It is likely that the number of women with previous GDM was slightly underestimated because of the under-diagnosis of GDM and the recall error. Despite the potential underestimation of GDM, interventions targeting women with GDM can have an appreciable population impact. It is worth noting that the estimates in our study differ from the estimates in the KPCO study; we estimated the proportion and the number of women aged 18 and older who had previous GDM only, whereas the KPCO study estimated the proportion of pregnancies with GDM among eligible pregnancies among KPCO members (2).

The economic impact of preventing the progression of diabetes among women with GDM would be great as well. A study in 1993 by Gregory et al estimated that a reduction in the development of type 2 diabetes among women with GDM by 10%, 25%, or 50% would yield a health care savings of $32 million, $140 million, or $331 million, respectively, over 10 years (17).

This study showed a high prevalence of modifiable risk factors among women with previous GDM and suggested a high potential for prevention. The study provides diabetes prevention and control programs with valuable information for targeting this high-risk group with appropriate intervention strategies. More attention to this issue is needed from health care providers and public health officials to encourage the promotion of healthy lifestyles during and following pregnancy. In part, the promotion involves more active dissemination and adoption of clinical practice guidelines, such as those of the Society of Obstetricians and Gynaecologists of Canada and the Canadian Society for Exercise Physiology (18).

It is estimated that approximately one third of people with diabetes are not aware of their diabetes status (19). Therefore, a proportion of women in the group with no diabetes and a proportion of women in the group with previous GDM only might have developed diabetes but were unaware of their diabetes status. Additionally, women with previous GDM only who have one or more modifiable risk factors are more likely to progress to overt diabetes than those without modifiable risk factors. The misclassification of diabetic status among women in the group with no diabetes, the progression of GDM to diabetes in the presence of risk factors, and the high prevalence of modifiable risk factors among the general public in the United States (e.g., 55.1% of women in the group without diabetes did not meet CDC physical activity recommendations) may partially explain why the estimated PORs for all modifiable risk factors among women with previous GDM only are not very high (ranging from 1.2 to 1.8).

A major limitation of this study is that diabetes, weight and height status, and behavioral factors are self-reported. However, reliability of self-reported diabetes is high (20). Furthermore, because we compared the prevalence among three diabetes groups, and the reporting errors are not likely to be varied by diabetes status, we would not expect this factor to influence the internal validity of this study.

## Figures and Tables

**Table 1:** Characteristics of Nonpregnant Women Aged 18 and Older by Diabetes Status, Behavioral Risk Factor Surveillance System, United States, 2003

Characteristic	No Diabetes, Weighted %	Previous Gestational Diabetes Only, Weighted %	Current Diabetes, Weighted %	Design-based F Test *P* Value
**Age, y**				
18-44	39.1	59.0	11.6	<.001
45-64	42.7	37.0	49.5	
≥65	18.2	4.0	38.9	
**Race and ethnicity**				
Non-Hispanic white	71.3	61.3	61.7	<.001
Non-Hispanic black	10.0	8.4	17.0	
Non-Hispanic Asian	2.3	3.6	1.9	
Native Hawaiian or Other Pacific Islander, non-Hispanic	0.4	0.4	0.4	
American Indian or Alaskan Native, non-Hispanic	0.9	1.4	1.7	
Other race, non-Hispanic	0.7	0.6	0.7	
Multiracial, non-Hispanic	1.4	2.1	1.8	
Hispanic	12.9	22.2	14.9	
**Education**				
Less than high school diploma	11.4	16.0	23.0	<.001
High school or general equivalency diploma	30.7	25.8	36.3	
Some college or technical school	28.3	30.0	25.0	
College or technical school degree	29.7	28.1	15.7	
**Annual household income, $**				
<15,000	12.9	15.4	28.7	<.001
15,000-24,999	18.7	17.1	25.2	
25,000-34,999	14.0	13.1	14.7	
35,000-49,999	16.8	16.9	14.3	
≥50,000	37.7	37.5	17.1	

**Table 2:** Modifiable Risk Factors Among Nonpregnant Women Aged 18 and Older, Behavioral Risk Factor Surveillance System, United States, 2003

Modifiable Risk Factor	Prevalence, % (95% CI)	Crude POR (95% CI)	Adjusted POR^a^ (95% CI)
**No leisure-time physical activity**			
No diabetes	25.7 (25.3-26.1)	Ref	Ref
Previous GDM^b^ only	32.0 (27.8-36.1)	1.4 (1.1-1.6)	1.4 (1.2-1.7)
Current diabetes	41.6 (39.9-43.2)	2.1 (1.9-2.2)	1.4 (1.3-1.5)
**Not meeting CDC physical activity recommendation^b^**			
No diabetes	55.1 (54.7-55.6)	Ref	Ref
Previous GDM only	57.4 (53.3-61.4)	1.1 (0.9-1.3)	1.2 (1.0-1.4)
Current diabetes	70.9 (69.3-72.5)	2.0 (1.8-2.1)	1.4 (1.3-1.6)
**Current smoker**			
No diabetes	20.0 (19.6-20.4)	Ref	Ref
Previous GDM only	20.1 (17.1-23.2)	1.0 (0.8-1.2)	0.9 (0.8-1.1)
Current diabetes	17.5 (16.2-18.8)	0.9 (0.8-0.9)	1.0 (0.9-1.1)
**Overweight (body mass index ≥25.0 kg/m^2^)**			
No diabetes	49.0 (48.5-49.5)	Ref	Ref
Previous GDM only	62.2 (58.3-66.0)	1.7 (1.5-2.0)	1.8 (1.6-2.2)
Current diabetes	80.5 (79.0-82.1)	4.3 (3.9-4.8)	3.4 (3.1-3.7)
**Obese (body mass index ≥30.0 kg/m^2^)**			
No diabetes	20.0 (19.6-20.4)	Ref	Ref
Previous GDM only	29.4 (25.7-33.1)	1.7 (1.4-2.0)	1.7 (1.4-2.1)
Current diabetes	51.2 (49.4-53.0)	4.2 (3.9-4.5)	3.6 (3.4-3.9)
**Fruit and vegetable consumption <5 servings per day**			
No diabetes	27.7 (27.3-28.2)	Ref	Ref
Previous GDM only	24.1 (20.5-27.6)	0.8 (0.7-1.0)	0.9 (0.7-1.1)
Current diabetes	31.3 (29.6-33.0)	1.2 (1.1-1.3)	1.1 (1.0-1.2)

POR indicates prevalence odds ratio; CI, confidence interval; Ref, reference group; CDC, Centers for Disease Control and Prevention; GDM, gestational diabetes mellitus.

a Adjusted for age, race and ethnicity, and education.

b 30 minutes of moderate-intensity physical activity per day on 5 or more days per week or 20 minutes of vigorous physical activity per day on 3 or more days per week, or both.
